# Bilateral Ovarian Hemangiomas Presenting With Abnormal Uterine Bleeding: A Rare Case Report

**DOI:** 10.1002/ccr3.70546

**Published:** 2025-05-30

**Authors:** Ensiyeh Bahadoran, Fatemeh SamieeRad

**Affiliations:** ^1^ Cellular and Molecular Research Center Research Institute for Prevention of Non‐Communicable Disease, Qazvin University of Medical Sciences Qazvin Iran; ^2^ Department of Pathobiology Faculty of Medical School, Qazvin University of Medical Sciences Qazvin Iran

**Keywords:** abnormal uterine bleeding, genital neoplasms female, histopathology, ovarian hemangioma, vascular tumors

## Abstract

Ovarian hemangiomas are rare benign vascular tumors that can present with abnormal uterine bleeding and mimic malignant ovarian lesions. Imaging may be inconclusive, and histopathology remains essential for diagnosis. This case highlights the importance of considering ovarian hemangiomas in the differential diagnosis of pelvic masses to ensure appropriate management.

## 1 Introduction

Hemangiomas are vascular tumors that are benign. Hemangiomas of the female genital tract are relatively rare [1]. Despite being a highly vascular organ, ovarian hemangiomas are uncommon, with less than 60 cases documented [2]. Payne et al. first identified ovarian hemangioma in 1869 in a female patient aged 25 who had bilateral ovarian hemangioma and abdominopelvic hemangiomatosis. The cases reported in the literature span an age range of 4 months to 81 years [3].

Frequently, they are found coincidentally at autopsy or as accidental findings following surgery. Some may have presentations like acute abdominal pain, torsion‐related abdominal pain, mass effect‐induced abdominal enlargement, ascites, thrombocytopenia, and an elevated level of CA125 [4, 5].

Although the precise etiology remains unknown, hypotheses have been proposed for the formation of ovarian hemangiomas. The first is the hormonal hypothesis. In this manner, cyclical changes in the ovaries during the reproductive years cause hyperestrogenism (linked to stromal hyperthecosis or hyperplasia), or excessive androgen production may promote growth in endothelial cells and cause vascular development. Another hypothesis suggests that stromal luteinization results from the presence of an expansile ovarian hemangioma. The steroid hormones made up of these luteinized stromal cells, primarily androgens, are subsequently converted into estrogens in adipose tissue, stimulating the endometrium. Moreover, factors such as infection, pregnancy, and hormonal changes can interfere with vascular development [6, 7].

Here, we report a case of ovarian hemangioma in a 42‐year‐old female patient who presented with abnormal uterine bleeding (AUB), aiming to contribute to the understanding of this rare entity and highlight its clinical features and differential diagnoses.

## 2 Case History/Examination

A 42‐year‐old female presented with AUB. She was a mother of three with no history of miscarriage or past medical diseases. In physical examination, the patient appeared well with stable vital signs. Abdominal examination revealed no tenderness or palpable mass. A speculum examination showed a normal cervix with no visible lesions. There were no signs of ascites or significant lymphadenopathy. Laboratory findings, including complete blood count (CBC), coagulation profile, and liver and renal function tests, were within normal ranges.

## 3 Investigations, Treatment and Differential Diagnosis

Pelvic sonography revealed a hyperechoic area measuring 106 × 82 mm in the anatomical location of the uterus, which had mild internal vascularity on color Doppler examination.

Subsequent magnetic resonance imaging (MRI) revealed a large, lobulated, heterogeneous signal mass lesion measuring 105 × 80 × 105 mm located in the left anterior aspect of the pelvic cavity. The lesion demonstrated areas of diffusion restriction on diffusion‐weighted imaging (DWI) and apparent diffusion coefficient (ADC) mapping, suggestive of either a left ovarian lesion (Ovarian‐Adnexal Reporting and Data System [ORADS] 5) or a punctuated subserosal myoma (International Federation of Gynecology and Obstetrics [FIGO] classification 7), with a possibility of sarcomatous changes. The CA125 level was measured and was 9.14 U/mL (Normal Level < 35 U/mL).

**FIGURE 1:**
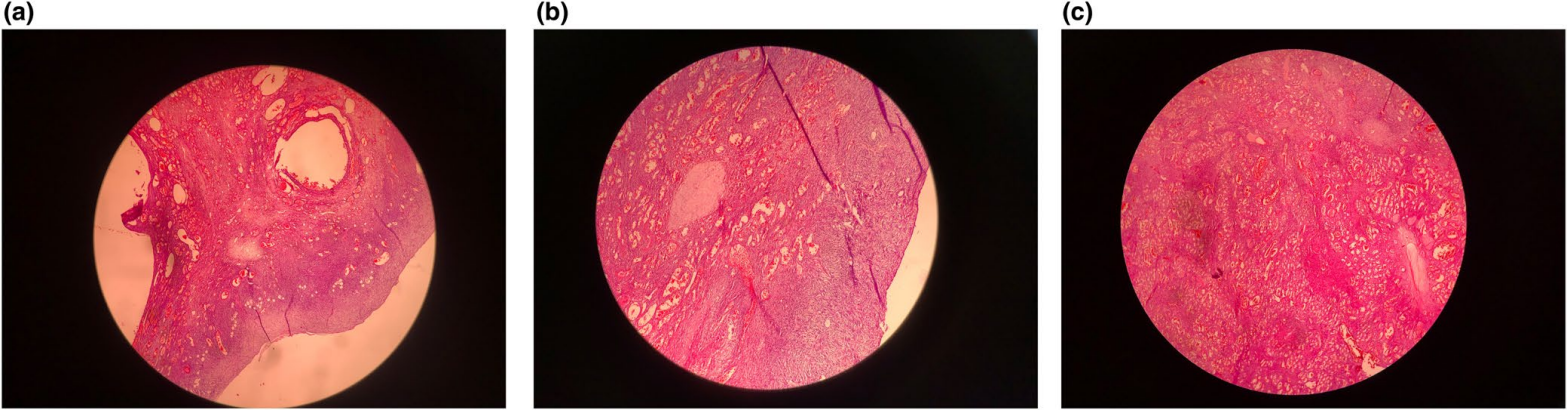
(a) The ovarian cortex with primary follicles of hemangioma vessels. 4×, Hematoxylin & Eosin staining. (b) The ovarian cortex contains primordial follicles and abundant blood vessels. 10×, Hematoxylin & Eosin staining. (c) Multiple blood vessels of hemangioma. 10×, Hematoxylin & Eosin staining.

Additional findings included uterine myomas: a right anterior subserosal myoma measuring 42 × 38 × 40 mm (FIGO classification 5), and an anterior subserosal myoma measuring 18 × 18 mm (FIGO classification 5). Multiple Nabothian cysts were also observed in the cervix. The right ovary appeared normal, but the left ovary could not be visualized. Macroscopically, the external aspect of both mildly enlarged ovaries was red or purplish. Cut sections show a finely spongy honeycomb pattern and dark brown dots. Histopathological evaluation revealed the presence of bilateral ovarian hemangiomas (Figure 1a–c).

The main differentials were lymphangioma, monodermal teratoma with prominent vascular components, and angiosarcoma [8, 9].

## 4 Outcome and Follow‐Up

The patient underwent a total abdominal hysterectomy with bilateral salpingo‐oophorectomy. Postoperative histopathological examination unexpectedly revealed bilateral ovarian hemangiomas as an incidental finding, with no evidence of malignancy. The postoperative level of CA125 decreased to 1.58 U/mL. The patient had an uneventful recovery and was discharged on postoperative Day 3. At a three‐month follow‐up, she reported complete resolution of AUB symptoms and no postoperative complications. The patient remains under routine gynecological surveillance.

## 5 Discussion

Hemangiomas are benign vascular tumors that develop when vascular development fails, especially during the canalizing phase, leaving behind unusual vascular channels. According to the assessment of the size of the blood vessels generated, these three varieties are the cavernous, capillary, and mixed types [2, 10]. In ovarian hemangioma, they manifest as cavernous, capillary, or mixed type, with a majority of cavernous type, in contrast to the rest of the body, where capillary hemangiomas are more common [7]. They consist of dilated, blood‐filled, often thin‐walled vessels that range in size from small to large and are coated with a single layer of endothelial cells that have been flattened. There may be calcification, hemorrhage, inflammation, and hemosiderin deposits in the stroma [7]. Due to their fragile endothelial layers, they are prone to hemorrhage or ascites [11]. On a macroscopic level, these lesion dimensions range from 5 mm to 24 cm [2]. They are mainly present unilaterally and on the circumference of the ovary [12]. In our case, it occurred bilaterally; the ovarian cortex contained primordial follicles, and abundant blood vessels of hemangioma indicated the diagnosis of mixed hemangioma.

The differential diagnoses can vary. The ovarian sclerosing stromal tumor exhibits a distinctive pattern with hypocellular regions of highly collagenous or edematous tissue separating the pseudolobulation of cellular sections. Large, thin‐walled vessels with variable degrees of sclerosis are mixed with spindled and lipid‐containing round to oval cells in cellular regions [13]. Microcystic stromal tumors are benign mixed epithelial and mesenchymal tumors that have three components: solid cellular areas, microcysts, and fibrous stroma. The fibrous stroma contains medium‐sized, monotonous, round‐to‐ovoid tumor cells with bland‐looking nuclei [14]. Another differential diagnosis is angiosarcoma, which is characterized by enhanced mitotic activity, necrosis, bleeding, papillary endothelial tufting, and noticeable cytological atypia [9].

Tubo‐ovarian masses, twisted ovarian cysts, and chocolate cysts are additional differentials of ovarian hemangiomas that can be pathologically differentiated from vascular growth, lymphangiomas, and monodermal teratomas with a substantial vascular component. Although ovarian teratomas usually do not have vascular elements, bilateral ovarian teratomas with a hemangiomatosis component have been reported; these can be distinguished from pure hemangiomas by the presence of respiratory epithelium [15]. The existence of red blood cells and the absence of pale eosinophilic secretion within vessels can rule out a diagnosis of lymphangiomas [3, 16]. Thrombocytopenia (Kasabach Merritt syndrome), ascites, and pleural effusion (pseudo‐Meigs' syndrome), and increased CA 125 that resembles ovarian surface epithelial malignancies have also been documented in cases of ovarian hemangiomas [3]. It is important to note that this hemangioma was an incidental finding in our case, as the patient presented with abnormal uterine bleeding, which was more likely attributed to a uterine myoma. Neither the clinician nor the radiologist had initially considered hemangioma in their differential diagnosis. Therefore, in this case, the diagnosis was established microscopically, and the diagnostic challenge was addressed with histopathological examination.

Color Doppler sonography and magnetic resonance imaging can be used for diagnosis [17]. However, histological examination confirms the presence of abnormal vascular channels lined by endothelial cells. The preferred course of treatment is surgical excision of the affected area. For young women, ovarian‐sparing surgery is advised [12].

## 6 Conclusion

In conclusion, ovarian hemangiomas are rare, benign tumors that can cause symptoms like AUB. Diagnosis is typically made through imaging and confirmed with histology. While their exact cause is unclear, surgical excision is the preferred treatment, with ovarian‐sparing surgery recommended for young women. This case emphasizes the importance of considering ovarian hemangiomas in the differential diagnosis of pelvic masses and AUB.

## Author Contributions

**Ensiyeh Bahadoran:** investigation, supervision, writing – original draft, writing – review and editing. **Fatemeh SamieeRad:** data curation, methodology, visualization, writing – review and editing.

## Ethics Statement

The authors have nothing to report.

## Consent

Written informed consent was obtained from the patient to publish this report in accordance with the journal's patient consent policy.

## Conflicts of Interest

The authors declare no conflicts of interest.

## Data Availability

All required data has been described in the article.

## References

[1] F. Gücer , F. Özyılmaz , P. Balkanlı‐Kaplan , N. Mülayim , and Ö. Aydın , “Ovarian Hemangioma Presenting With Hyperandrogenism and Endometrial Cancer: A Case Report,” Gynecologic Oncology 94, no. 3 (2004): 821–824. 15350380 10.1016/j.ygyno.2004.06.021

[2] D. Nakuci , E. Kola , E. Horjeti , et al., “Ovarian Hemangioma Presented as an Incidental Ovarian Mass: A Rare Case Report Along With Literature Review,” Archives of Clinical and Medical Case Reports 4, no. 5 (2020): 760–765.

[3] M. Dahal , P. Upadhyaya , P. Adhikari , D. Karki , and N. Regmi , “Ovarian Hemangioma: A Rare Entity,” International Journal of Reproduction, Contraception, Obstetrics and Gynecology 7, no. 6 (2018): 2490–2492.

[4] R. S. Huang , M. Covinsky , and S. Zhang , “Bilateral Ovarian Capillary Hemangioma With Stromal Luteinization and Hyperandrogenism,” Annals of Clinical and Laboratory Science 43, no. 4 (2013): 457–459. 24247806

[5] Y. Kaneta , R. Nishino , K. Asaoka , K. Toyoshima , K. Ito , and H. Kitai , “Ovarian Hemangioma Presenting as Pseudo‐Meigs' Syndrome With Elevated CA125,” Journal of Obstetrics and Gynaecology Research 29, no. 3 (2003): 132–135. 12841694 10.1046/j.1341-8076.2003.00088.x

[6] J. DiOrio and L. C. Lowe , “Hemangioma of the Ovary in Pregnancy: A Case Report,” Journal of Reproductive Medicine 24, no. 5 (1980): 232–234. 7401054

[7] K. Ziari and K. Alizadeh , “Ovarian Hemangioma: A Rare Case Report and Review of the Literature,” Iranian Journal of Pathology 11, no. 1 (2016): 61–65. 26870145 PMC4749197

[8] H. Itoh , T. Wada , K. Michikata , et al., “Ovarian Teratoma Showing a Predominant Hemangiomatous Element With Stromal Luteinization: Report of a Case and Review of the Literature,” Pathology International 54, no. 4 (2004): 279–283. 15028031 10.1111/j.1440-1827.2004.01621.x

[9] P. A. Gehrig , W. C. Fowler, Jr. , and R. A. Lininger , “Ovarian Capillary Hemangioma Presenting as an Adnexal Mass With Massive Ascites and Elevated CA‐125,” Gynecologic Oncology 76, no. 1 (2000): 130–132. 10620457 10.1006/gyno.1999.5648

[10] A. Emami , E. Bahadoran , and F. SamieeRad , “Cavernous Hemangioma of Corpus Imitating Endometrial Polyp in a Young Non‐Pregnant Woman: A Case Report Study,” Clinical Case Reports 12, no. 1 (2024): e8413. 38188844 10.1002/ccr3.8413 PMC10766558

[11] M. L. A. Miranda and E. I. Valencia , “A Life‐Threatening Benign Vascular Lesion of the Uterus, Cavernous Hemangioma: A Case Report,” Philippine Journal of Obstetrics and Gynecology 47, no. 5 (2023): 283–290.

[12] M. Hafsi , M. Moussi , S. Najar , F. Dridi , S. Maroua , and M. Mourali , “Ovarian Hemangioma: Differential Diagnosis of Ovarian Cancer,” International Journal of Surgery Case Reports 116 (2024): 109431. 38394939 10.1016/j.ijscr.2024.109431 PMC10944122

[13] M. Atram , S. Sharma , and N. Gangane , “Sclerosing Stromal Tumor of the Ovary,” Obstetrics & Gynecology Science 57, no. 5 (2014): 405. 25264533 10.5468/ogs.2014.57.5.405 PMC4175603

[14] Y. Zhang , L. Tao , C. Yin , et al., “Ovarian Microcystic Stromal Tumor With Undetermined Potential: Case Study With Molecular Analysis and Literature Review,” Human Pathology 78 (2018): 171–176. 29458068 10.1016/j.humpath.2018.02.012

[15] M. Singh , P. Subedi , B. Adhikari , et al., “Ovarian Hemangioma: A Rare Encounter,” Clinical Case Reports 12, no. 9 (2024): e9362. 39210922 10.1002/ccr3.9362 PMC11358698

[16] P. K. Sharma , A. Mohanakrishnan , A. P. Amir , A. Sekar , and S. S. Amir , “Isolated Unilateral Ovarian Cystic Lymphangioma: A Case Report,” Radiology Case Reports 19, no. 9 (2024): 3732–3739. 38983278 10.1016/j.radcr.2024.05.042 PMC11231512

[17] G. Cormio , G. Loverro , M. Iacobellis , L. Mei , and L. Selvaggi , “Hemangioma of the Ovary. A Case Report,” Journal of Reproductive Medicine 43, no. 5 (1998): 459–461. 9610472

